# 

**Published:** 1908-04

**Authors:** 


### BOOKS RECEIVED.

The Diagnosis and Treatment of Pulmonary Tuberculosis. By Francis M. Pottenger, A.M., M.D., Medical Director of the Pottenger Sanatorium for Diseases of the Lungs and Throat, Monrovia, Cal.; Professor of Clinical Medicine in the Medical Department, University of Southern California. Octavo, pp. 377. New York: William Wood & Co. 1908. (Cloth, $3.50.)

History of the Study of Medicine in the British Isles. The Fitz-Patrick Lectures for 1905-6, delivered before the Royal College of Physicians of London. By Norman Moore, M.D., Cantab. Fellow of the Royal College of Physicians; Physician to St. Bartholomew's Hospital. Octavo, pp. 202. Oxford: Clarendon Press. 1908.

A Textbook of Surgical Anatomy. By William Francis Campbell, M.D., Professor of Anatomy in the Long Island College Hospital. Octavo, pp. 675. Illustrated. Philadelphia and London: W. B. Saunders Co. 1908. (Cloth, $5.00.)

The Blues (Splanchnic Neurasthenia). Causes and Cure. By Albert Abrams, A.M., M.D., (Hiebelberg), F.R.M.S., Consulting Physician, Denver National Hospital for Consumptives. 12 mo, pp. 287. Illustrated. Third Edition. New York: E. B. Treat & Co. 1908. (Cloth, $1.50.)

Bradycardia and Tachycardia, with complete English abstracts and foreign bibliography. Part II. in a series of monographs on the Symptomatology and Diagnosis of Disorders of Respiration and Circulation. By Prof. Edmund Von Neusser, Professor of the Second Medical Clinic, Vienna; Associate Editor of Nothnagel's Practice of Medicine. Authorised English Translation by Andrew MacFarlane, M.D., Professor of Medical Jurisprudence and Physical Diagnosis, Albany Medical College. 12 mo, 150 pages. New York: E. B. Treat & Co. 1908. (Cloth, $1.25.)

Merck's 1907 Index. An Encyclopedia for the Chemist, Pharmacist and Physician. Third Edition. New York: Merck & Co.

Report of the Commissioner of Education for the Year ending June 30, 1906. Vol. 2. Washington: Government Printing Office.

Transactions of the Thirteenth annual meeting of the American Laryngological, Rhinological and Otological Society, held at New York, May 30 and 31, and June 1, 1907. W. H. Haskin, M.D., Secretary.

Public Health and Marine Hospital Service of the United States. Walter Wyman, M.D., Surgeon-General. Hygienic Laboratory. Milk and its Relation to the Public Health. By various authors. Washington: Government Printing Office.

Annual Report of the Surgeon-General of the Public Health and Marine Hospital Service of the United States. For the fiscal year, 1907. Walter Wyman, M.D., Surgeon-General. Washington: Government Printing Office.

Progressive Medicine, Vol. X, March, 1908. A Quarterly Digest of Advances, Discoveries and Improvements in the Medical and Surgical Sciences. Edited by Hobart Amory Hare, M.D., Professor of Therapeutics and Materia Medica in the Jefferson Medical College of Philadelphia. Octavo, 284 pages. Lea Brothers & Co., Publishers, Philadelphia and New York. Per annum in four cloth-bound volumes, $9.00; in paper binding, $6.00, carriage paid to any address.

